# Recruitment of older adults to three preventative lifestyle improvement studies

**DOI:** 10.1186/s13063-018-2482-1

**Published:** 2018-02-20

**Authors:** Robin Chatters, Louise Newbould, Kirsty Sprange, Daniel Hind, Gail Mountain, Katy Shortland, Lauren Powell, Rebecca Gossage-Worrall, Tim Chater, Anju Keetharuth, Ellen Lee, Bob Woods

**Affiliations:** 1 School of Health and Related Research, The University of Sheffield, Regent Court, Regent Street, Sheffield, S1 4DA UK; 2 Faculty of Medicine and Health Sciences, Queen’s Medical Centre, Nottingham, NG7 2UH UK; 3 Sheffield NIHR Clinical Research Facility, O Floor, Royal Hallamshire Hospital, Glossop Road, Sheffield, S10 2JF UK; 4 DSDC Wales, Bangor University, Normal Site, Bangor, LL57 2PZ UK; 5 Research Fellow Mental Health and Addiction Research Group Department of Health Sciences ARRC Building University of York, Heslington, York YO10 5DD UK

**Keywords:** Complex interventions, Randomised controlled trials, Study design and best practice

## Abstract

**Background:**

Recruiting isolated older adults to clinical trials is complex, time-consuming and difficult. Previous studies have suggested querying existing databases to identify appropriate potential participants. We aim to compare recruitment techniques (general practitioner (GP) mail-outs, community engagement and clinician referrals) used in three randomised controlled trial (RCT) studies assessing the feasibility or effectiveness of two preventative interventions in isolated older adults (the Lifestyle Matters and Putting Life In Years interventions).

**Methods:**

During the three studies (the Lifestyle Matters feasibility study, the Lifestyle Matters RCT, the Putting Life In Years RCT) data were collected about how participants were recruited. The number of letters sent by GP surgeries for each study was recorded. In the Lifestyle Matters RCT, we qualitatively interviewed participants and intervention facilitators at 6 months post randomisation to seek their thoughts on the recruitment process.

**Results:**

Referrals were planned to be the main source of recruitment in the Lifestyle Matters feasibility study, but due to a lack of engagement from district nurses, community engagement was the main source of recruitment. District nurse referrals and community engagement were also utilised in the Lifestyle Matters and Putting Life In Years RCTs; both mechanisms yielded few participants. GP mail-outs were the main source of recruitment in both the RCTs, but of those contacted, recruiting yield was low (< 3%). Facilitators of the Lifestyle Matters intervention questioned whether the most appropriate individuals had been recruited. Participants recommended that direct contact with health professionals would be the most beneficial way to recruit.

**Conclusions:**

Recruitment to the Lifestyle Matters RCT did not mirror recruitment to the feasibility study of the same intervention. Direct district nurse referrals were not effective at recruiting participants. The majority of participants were recruited via GP mail-outs, which may have led to isolated individuals not being recruited to the trials. Further research is required into alternative recruitment techniques, including respondent-driven sampling plus mechanisms which will promote health care professionals to recruit vulnerable populations to research.

**Trial registration:**

International Standard Randomised Controlled Trial Registry, ID: ISRCTN28645428 (Putting Life In Years RCT). Registered on 11 April 2012;

International Standard Randomised Controlled Trial Registry, ID: ISRCTN67209155 (Lifestyle Matters RCT). Registered on 22 March 2012;

ClinicalTrials.gov, ID: NCT03054311 (Lifestyle Matters feasibility study). Registered retrospectively on 19 January 2017.

## Background

The importance of recruiting to randomised control trials (RCTs) is well established in the academic literature [1–3]. Insufficient recruitment can result in statistically significant findings not being reported where a true difference does exist as well as it having negative cost implications [4–7]. A recent systematic review of publicly funded RCTs found that only 31% (38/122) of trials reached their original sample size, and that 34% (13/38) of these required an extension [7]. In particular, evaluations of complex interventions (those that consist of various distinct, but interacting, elements) are at particular risk of being undermined due to issues with recruitment and delivery of the intervention [8].

Studies involving older adults are at risk of certain barriers to recruitment, such as identification of potential individuals, informed consent and physical access issues, with the gaining of informed consent being negatively affected by poor health and concerns around being randomised to the control group [1, 9]. Such barriers cause participation biases and can lead to the recruited population not being the intended recipients of the intervention (i.e. healthy volunteer bias) [10]. Previous studies have aimed to identify adaptations that can be made to recruitment strategies in order to improve participation rates, finding that opt-out methods, telephone reminders and open designs (i.e. where the participant knows which arm of the trial they will receive) are beneficial, but are not always possible in clinical trials where ethical considerations and blinding of participants is often methodologically important [11]. Survey data from clinical trials units in the UK have identified methods used to encourage recruitment including patient contact, recruiter support and incentives [12].

Database recruitment, where participants are identified from health service records, has been proposed as an option for recruiting individuals to clinical trials, with advantages including being able to identify individuals easily and recruit in a time-effective way via mail-out [6]. Disadvantages include the inability to identify individuals with acute conditions, and confidentiality issues [6]. Sending mail-outs to potential participants contrasts with studies reporting that individuals value good contact with the research team prior to consenting [9]. However, mail-outs have been used successfully to recruit individuals to previous studies, often (but not always) to schedule and above target [13–17]. One such study (the Food and Immunity Trial, or ‘FiT study’), aimed to recruit older adults to a preventative dietary intervention study, found that recruitment of participants through database via general practitioner (GP) surgeries was successful; other recruitment techniques, including direct clinician referral, did not prove as successful [16].

Qualitative evidence obtained from researchers and participants is important to assess the acceptability of recruitment methods. Previous qualitative evidence has found that researchers prefer the use of targeted mail-outs compared to clinical referral as it allows more accurate prediction of recruitment rates during the trial [18]. RCTs with embedded qualitative studies can assess barriers to recruitment, identifying changes to the design and conduct of a trial which improve consent rates [19].

This paper aims to provide a basis for planning recruitment of similar participants in future trials, presenting quantitative data from three studies and qualitative data from one study. The three study interventions were preventative and targeted older adults through a group-based occupational therapy intervention (the Lifestyle Matters feasibility trial [20], the Lifestyle Matters RCT [21]) and telephone friendship groups (the Putting Life In Years RCT [22]).

## Methods

### Overview of projects

#### Lifestyle Matters feasibility study and Lifestyle Matters RCT

The Lifestyle Matters feasibility study was undertaken between 2004 and 2005 in a city in the north of England and aimed to assess the feasibility of recruiting older adults to the Lifestyle Matters intervention, which involved participants aged 65 + years attending weekly group meetings over 8 months facilitated by two trained staff and the offer of attending four one-to-one sessions with one of the facilitators to pursue individual goals [23]. All recruited participants received the intervention. During the group meetings participants were encouraged to think about, and engage in, discussion and activities related to general health and wellbeing as part of everyday life. Each group was encouraged by the facilitators to explore topics through discussion and then to explore this further in practice through activities and outings. The individual sessions were designed to offer the participant time and space to address individual needs, ideas or interests. The main objective of the intervention was to promote general health and wellbeing through long-term change.

Between 2011 and 2015, the Lifestyle Matters RCT was undertaken, where a total of 11 Lifestyle Matters programmes were delivered to those who were randomised to the intervention group in addition to usual care: six in a city in the north of England and five in rural North Wales [24]. Participants were recruited between August 2012 and April 2013; those randomised to the control group received usual care only. The RCT was influenced by lessons learnt from the feasibility study – recruitment techniques were adapted (discussed below) and the intervention was manualised and published following adaptations (i.e. changes to group session topics, schedules and exercises) [25]. The length of the intervention was shortened to 4 months in order to increase the feasibility of being able to potentially deliver the intervention within the National Health Service (NHS). A qualitative analysis of the long-term impact of the intervention has been published elsewhere [26].

#### Putting Life In Years RCT

Putting Life In Years was an RCT that, between June 2011 and December 2012, recruited older adults aged 75 + years to a study that provided the intervention arm with weekly group telephone calls facilitated by trained volunteers. The intervention was provided over two stages. Initially, one-to-one telephone conversations were arranged to allow the older person to be supported and prepared for the second stage, which involved group conversations with approximately five other participants over a 12-week period. Participants randomised to the control group received usual care only. The study is described in full elsewhere [27].

### Participant recruitment

Recruitment commenced in early 2004 for the Lifestyle Matters feasibility study, June 2011 for the Putting Life In Years RCT and December 2011 for the Lifestyle Matters RCT. The three studies utilised similar participant inclusion criteria and recruitment techniques which are presented in Table 1. Although the three studies aimed to recruit those who were socially isolated, an eligibility clause related to isolation was not included, as it was thought to be too restrictive, as such individuals may not identify as being such. Instead, recruitment was targeted to those who were isolated. The feasibility study initially asked district nurses to stimulate interest among potentially suitable individuals; it was thought that district nurses would be in an ideal position to identify isolated individuals who would benefit most from the intervention. Potential participants were asked to telephone the university to find out more information about the study. A community engagement process was also developed (reasons for this presented in results) which involved presentations and taster sessions to local health forums and to community groups in the locality to stimulate interest.

**Table 1:** Inclusion criteria and recruitment techniques utilised in the three studies

Study	Inclusion criteria	Recruitment techniques
Lifestyle Matters RCT	• Aged 65 years (Lifestyle Matters) or 75 years (Putting Life In Years) and over • Reasonable cognitive function (a score of 0–7 on 6-item Cognitive Impairment Test) • Living independently in sheltered accommodation, alone or with others in specific areas of Sheffield (Putting Life In Years/Lifestyle Matters) and Bangor (Lifestyle Matters) • Able to converse in English	• Referrals by district nurses • Community engagement • GP mail-outs • South Yorkshire Cohort^a^ (Putting Life In Years only)
Putting Life In Years RCT		
Lifestyle Matters feasibility study	• MMSE (Mini-Mental State Examination) score over 18 • Geriatric Depression Scale score not indicating severe depression • Aged 60 + years	• Referrals by district nurses • Community engagement

^a^South Yorkshire Cohort is a study that has recruited patients from GP surgeries in the South Yorkshire area. Studies can use the cohort to recruit individuals

*GP* general practitioner, *RCT* randomised controlled trial

GP mail-outs were utilised for the Lifestyle Matters RCT and Putting Life In Years RCT. Local GP surgeries were identified through the Primary Care Research Network (PCRN) to ascertain their interest. Interested GPs were often larger practices that were research active and able to identify resources to undertake large mail-outs. Each interested GP surgery was then approached by a member of the study team to arrange a time to discuss the study and arrange site set up. It was requested that GPs send potential eligible participants (patients registered to their surgery who met the age inclusion criteria for the study) an invitation letter and study leaflet along with a pre-paid response card. Potential participants who were interested completed the response card, indicating whether or not they met the initial eligibility criteria as indicated on the card and returning it to the study team. On receipt, the study team telephoned the individual to arrange a screening visit.

Regardless of which method used to approach the individual, a research assistant (RA) visited each potential participant at home for a screening visit. During the visit, the study was discussed with the participant before undertaking the Six-item Cognitive Impairment Test (6CIT) to test for cognitive impairment; those who obtained a score of 8 or more were deemed ineligible and advised to visit their GP [28]. Those deemed eligible could then either consent to the study and complete the baseline measures, have more time to consider whether or not to take part in the study and be contacted again by the research team, or decline to participate. Consenting participants were randomised to either the control or the intervention group.

For the Putting Life In Years RCT, mail-outs were also sent via an existing cohort study (the South Yorkshire Cohort) to individuals who were willing to be contacted regarding future research [29]. In January 2013, recruitment to the Putting Life In Years RCT was halted early due to issues with delivering the intervention by the local branch of a national charity, despite meeting internal feasibility targets for the recruitment and retention of participants [22].

### Data collection

#### Quantitative

During the recruitment phase of each of the studies, records were kept regarding how participants were recruited and the number of participants who consented to take part per recruitment method. In addition, for the two studies utilising GP mail-outs (the Lifestyle Matters RCT and the Putting Life In Years RCT), records were kept regarding the number of mail-outs sent per GP surgery.

#### Qualitative

The Lifestyle Matters RCT was the only study to explore recruitment through qualitative interviews. Both trial participants and facilitators were given the opportunity to consent to be contacted about the interviews at the start of the RCT. Consent was then obtained to undertake interviews with facilitators and trial participants in late 2013/early 2014, 6 months after the start of the intervention. Trial participants were selected via purposeful sampling across the 11 groups, aiming to interview individuals with a mix of age, sex, living arrangements (alone/with other), occupation and number of Lifestyle Matters group sessions attended. Once the Lifestyle Matters group had completed its final monthly meeting, the researchers contacted the selected participants by telephone to invite them to take part in an interview. A date, time and venue were agreed and a letter confirming these details was sent to the participant prior to the interview. Participants were contacted again by telephone the day before the interview to confirm arrangements and that they were still happy to take part. Interviews were undertaken by Dr. Sarah Cook (SC) PhD (fidelity lead) and KS (trial manager of Lifestyle Matters RCT), both having extensive experience in qualitative interviews and analysis. Interviews were undertaken at each participant’s home address, with no one else present. Apart from telephoning the participants prior to the interview to arrange a convenient date and location, there was no relationship between the researchers and participants. Prior to the interview, participants were aware that the researchers were visiting to discuss their experience of the Lifestyle Matters project; participants would have been aware of the researchers interest in the project. Sixteen trial participants were approached for participation in the qualitative study, three participants declined and 13 participants consented to being interviewed. Interviews lasted between 14 and 71 min (median = 38 min). Table 2 presents the characteristics of the recruited participants.

**Table 2:** Demographics of Lifestyle Matters randomised controlled trial (RCT) participants interviewed

Participant ID	Age	Sex	SF-36 mental health score^a^	Lives with anyone?	Number of group sessions attended^b^	Last employment
Participant A	72	Male	90	Yes	15	Mobile library driver
Participant B	79	Female	85	Yes	1	Primary school teacher
Participant C	69	Female	90	No	14	Health support worker
Participant D	77	Female	75	Yes	13	Machine hand
Participant E	65	Male	70	No	15	Psychiatric staff nurse
Participant F	77	Male	70	No	15	Haulage contractor
Participant G	73	Female	50	No	14	Teacher
Participant H	92	Male	90	No	9	Building contractor
Participant I	88	Female	90	No	12	Unknown
Participant J	70	Female	90	Yes	9	Retail assistant
Participant K	69	Female	65	No	14	Unknown
Participant L	68	Female	55	No	10	Hairdresser
Participant M	72	Male	85	No	12	Sales director

^a^SF-36 mental health score at six months post randomisation, The SF-36 mental health dimension is scored on a scale from 0 (poor) to 100 (good) ^b^out of a possible 16 sessions

All four intervention facilitators were approached by telephone to ascertain their interest in participating in the interviews; all four agreed to participate and were interviewed by SC and KS on university premises in order to gain their perspective on the recruitment strategies. Demographics of facilitators were not collected, but all were female and had previously worked with adults with dementia. Although the posts were designated as non-professional, Band-4 NHS grade, two were registered occupational therapists, one was a qualified social worker and one had worked as a mental health advocate. One of the two facilitators located in Wales was fluent in Welsh as well as English. Interviewees would have been aware of the researcher’s invested interest in the trial, and their involvement in the running and implementation of the RCT. All four facilitators were interviewed at two time points at the end of recruitment waves 1 and 3. Interviews lasted between 56 and 95 min (median = 82 min).

All interviews with both participants and facilitators were audio-recorded, no one else was present at the time and field notes were not taken. Interviews were undertaken using semi-structured topic guides (one for trial participants and another for facilitators); the trial participant guide was reviewed by the trial Patient and Public Involvement (PPI) members during its development. Both topic guides aimed to identify perspectives towards the recruitment techniques utilised in the trial, focussing on (1) the acceptability of the methods used, (2) their thoughts on the apparent isolation of the recruited participants and (3) their thoughts on how recruitment to the trial could be improved. Interviewees were not provided with a definition of ‘isolation’; rather, they relied on their own thoughts and experiences regarding this. Following the interviews, transcripts were returned to participants and facilitators for comments and corrections. Repeat interviews were not carried out.

### Analysis

A framework approach was utilised in qualitative data analysis, which took a case study approach [30]. Framework Analysis is a five-stage process of: familiarisation, forming a thematic framework, indexing, charting and mapping and interpretation. The analysis was undertaken by the same two researchers (SC and KS). Firstly, both researchers independently familiarised themselves with the same sample of interview transcripts for facilitators and participants through reading and rereading, at which point saturation was confirmed. The same sample of transcripts were then coded for emergent themes independently by each researcher before being triangulated for cross-cutting and diverging themes between the participant sample or facilitator sample and across both samples. From this an initial thematic framework, consisting of major nodes and one level of ‘tree’ nodes, was developed by the two researchers. This framework was then applied to the same sample of transcripts. The researchers met to discuss any differences which were managed through consensus before finalising the framework. All transcripts were then coded by either SC or KS using the final index using NVivo 10 software. Matrices were developed, with one major node per matrix. These matrix charts were then examined for cross-cutting themes and patterns in the data were mapped to inform the final level of interpretation. In writing up the analysis, quotations that demonstrated the breadth of opinions were chosen. Participants did not provide feedback on the findings.

### Ethical considerations

For the qualitative aspect of the Lifestyle Matters RCT, all participants and facilitators provided written informed consent for participation in the study. Ethical approval for the main trial and this qualitative sub-study was granted by the South Yorkshire Research Ethics Committee (reference number 12/YH/0101). Transcripts were anonymised prior to data analysis in order to preserve confidentiality.

## Results

A total of 288 individuals were recruited to the Lifestyle Matters RCT, 28 to the Lifestyle Matters feasibility study and 157 to the Putting Life In Years RCT. Participants were recruited via various mechanisms (Table 3).

**Table 3:** Number of individuals recruited by recruitment method and study

Study	Target sample size	Total recruited	Recruitment method					
			GP mail-out (% of total recruited)	Referral^a^ (% of total recruited)	South Yorkshire Cohort (% of total recruited)	Other^b^ (% of total recruited)	Community engagement (% of total recruited)	Unknown (% of total recruited)
Lifestyle Matters feasibility study	N/A	28	N/A	0	N/A	3 (10.7)	25 (89.3)	0
Lifestyle Matters RCT	268	288	270 (93.8)	15 (5.2)	N/A	3 (1.0)	0	0
Putting Life In Years RCT	N/A^c^	157	136 (86.6)	3 (1.9)	11 (7.0)	5 (3.2)	0	2 (1.3)

^a^District nurse referrals, plus participants who were signposted from other services such as Sheffield 50 + or from a health and social care worker

^b^Recruitment through family member/friend or research staff in addition to participants seeing posters in libraries and other recruitment literature in the public domain, such as advertisements in their local newspaper

^c^The target sample size was 248; however, recruitment halted early (63% of target) due to issues relating to service provide capacity to deliver the intervention at scale

*GP* general practitioner, *N/A* not applicable, *RCT* randomised controlled trial

### Lifestyle Matters feasibility study

District nurse referrals did not yield any participants; this appeared to be primarily due to a lack of engagement in the study. In addition, requesting that participants telephoned the university to find out information about the study may have been an additional barrier for some individuals, excluding individuals with low confidence or hearing problems. As a result, a programme of community engagement activities were conducted which enabled the recruitment of 25 participants. Community engagement overcame communication barriers allowing participants to gain a ‘taste’ of the intervention, allowing 89.3% of the studies target to be recruited.

### Lifestyle Matters and Putting Life In Years RCTs

#### Quantitative results

Due to the issues with district nurse referrals in the feasibility study, GP mail-outs were used in addition to referrals in the Lifestyle Matters and Putting Life In Years RCTs, along with community engagement activities. In terms of numbers recruited, GP mail-outs proved to be the most effective recruitment method for these two studies, recruiting a high proportion of each RCT’s target number of participants. Out of the 18,331 study packs sent to eligible participants, the overall response rate from GP mail-outs was 2.3% (see Table 4). There was also variation between GP surgeries (between 0.3% and 5.4% of those approached were recruited). Community engagement did not result in any participants being recruited to the RCTs, and district nurse referrals resulted in very few participants being recruited. In similarity with the feasibility study, district nurses did not engage with the trial – nurses attended recruitment training sessions but did not recruit individuals during their routine clinical practice.

**Table 4:** Number of participants recruited by GP mail-outs per study

Study	Site	Number of GP surgeries	Number sent	Number recruited	% recruited (range of % between surgeries)
Lifestyle Matters RCT	North Wales	8	3705	129	3.5 (1.5–5.4)
	North England	7	5625	152	2.7 (0.6–3.7)
Putting Life In Years RCT	North England	19	9051	136	1.5 (0.3–3.3)

*GP* general practitioner, *RCT* randomised controlled trial

#### Qualitative results

##### Reflections on recruitment techniques

Participants in the Lifestyle Matters RCT reflected on the fact that, on receiving the letter, they were not sure what the focus of the intervention was. This may have had a negative effect on recruitment:

*‘I think if they had a bit more information in the initial letter... But all it said was that, you know, they were doing this research project…there was no information on what to really expect, I think there could have perhaps been a little bit more than that but apart from that I don’t think there was anything else’. (Participant E)*

Recruited participants found the mail-outs to be a satisfactory mode of recruitment. Nearly all participants who took part in the study had been contacted via their GP and this was generally considered a good method of identifying people due to the physician’s personal knowledge of their patients’ circumstances:

*‘Yes, that was a good way to contact people. I mean they know if anybody’s sort of lonely or housebound or, you know, or recently bereaved or anything like that’. (Participant B)*

Participants also considered that health and social care services would be expertly placed to identify appropriate individuals who would benefit from the programme:

*‘…so they might, you know, consult with social services, you know, what area do you have people who live on their own…’. (Participant L)*

Referrals via health and social care, attendance of research staff at local groups and advertising the studies via posters and newspapers did not yield many participants. Despite this, participants felt that these recruitment mechanisms could work. Advertising using leaflets or posters locally; for example, through community venues and services, was recommended by a number of participants as a good recruitment strategy. These individuals typically used these venues and stated that they regularly looked at notice boards for information on what was happening in their community. Advertisements posted through doors were also proposed which could be targeted through social housing:

*‘…put a note, in, in the church hall, where people use that hall a lot…or doctor’s places to let them know that there’s, that there’s this thing going on. And people do, people do because we, we always look at notice boards to see what is going off’. (Participant D)*

##### Apparent isolation of recruited participants

All four Lifestyle Matters intervention facilitators commented that the type of participant recruited was not what they expected. From their training they expected people who were isolated, somewhat fragile and stuck in a rut. These would be people who had become lonely and inactive after a major change in their lives or transition such as retirement, bereavement or a long-term illness, and were not confident at accessing groups and services. In contrast, they found that the majority of participants were confident, sociable, resourceful and busy:

*‘Everybody was mega-confident, you know… Everybody dying to talk, no issues kind of expressing themselves and so forth, really kind of confident’. (Facilitator 1)*

*‘...they already seem to have very effective ways of managing their lifestyle and, and keeping time for things that they want to do as well as things they have to do, so it might not have had a huge impact on them.’ (Facilitator 3)*

However, over the course of the programme, facilitators found that even those appearing confident and busy benefitted from the programme by trying new activities and reassessing priorities:

*‘...for some people they don’t wanna face things or there’s other things going on or they’ve just retired or it’s filling time and, you know, you just kind of cram your time with different activities or doing things and for them it was about thinking what’s important what’s useful for me what am I doing for other people?’ (Facilitator 2)*

Participants also reflected on their own isolation; many did not identify themselves as being either isolated or lonely, but these were the key traits propounded for who would benefit from this programme. For example, participants described potential participants as those who lived alone, had a recent bereavement, experienced a life-changing event, needed to get out more, had no family or did not see family very often:

*‘I think really it’s more for people on their own really than people that have got partners... I’m not lonely, I’m not on my own but there’s quite a few people that go there that are feeling lonely, their families are grown up and they don’t see much of them...’. (Participant D)*

*P: ‘Somebody’s who’s housebound or needing to get out or somebody who perhaps just been on their own or needs sort of rebuild their lives. I met someone her husband’s recently died and they’re wanting to get back in to things’. (Participant C)*

##### Improving recruitment in the Lifestyle Matters Study

Within the Lifestyle Matters RCT, participants and facilitators were asked their thoughts on improving recruitment. They focussed on not only improving the number of participants recruited to the study, but also improving the suitability of those who participated.

To increase the number recruited, some participants felt that their health services could have been more proactive in recommending the programme to them. One individual described how they picked up a leaflet about the study in their GP surgery, but had not received any direct encouragement from their GP to attend:

*‘Nobody in the medical or health professions encouraged me to do so it was my own initiative…’. (Participant G)*

A wide range of recruitment methods were suggested by participants to target isolated and lonely people living on their own whom they considered would benefit most from taking part in the Lifestyle Matters RCT. Participants felt that this would be isolated or lonely people living on their own. One participant recognised the difficulty of identifying such individuals:

*‘…but how do you find them? How do you find them? You know’. (Participant F)*

Facilitators suggested that as well as GPs recruiting participants, individuals could be recruited by community groups and health and social care workers:

*‘I think, yes, support services, doctors, health professionals that kind of thing and it is difficult because I think we tried that for referrals from occupational therapists and not a lot came out of it and I don’t know whether that was understanding of the programme or the particular level that it’s pitched at the moment, I d-don’t know’. (Facilitator 2)*

*‘Suppose if they were in the communities then they could be, you know, posters up and some of the other groups that are out there already, you know, WI could keep an eye out for people that they think might benefit and more informal contacts, erm, but then the organisation of that gets more tricky if there was one overseer of the project. I think it’s different if they are just running in the community and, you know, taking ownership of them and it was a less informal group it might be easier for people.’ (Facilitator 3)*

An interesting approach suggested by participants was using word of mouth; for example, people who had completed the programme recommending it to other people as peers (i.e. ‘snowballing’). This was also suggested by a facilitator. This implies an element of trust which would help people make up their mind to take part if they either knew someone who had done the programme or they could speak to someone first-hand about their experience:

*‘…I think the only way you would encourage other people to go is by someone of my age who’d attended a group actually going and talking to those people, because it’s very much a “suck it and see” situation, what works for me doesn’t work for someone else’. (Participant A)*

*‘...if it was a person that already knew about the programme and thought that this could be really useful for you and they are talking about it they might be more able to get a foot in the door perhaps.’ (Facilitator 3)*

Facilitators felt that participants’ attributes were important to take into account when deciding the composition of the groups. Facilitators felt that individuals from a similar life stage, situation or community should be put together in the same group:

*‘I wondered, it’s less about age but more about life stage, erm, I don’t know for some people if you’re better kind of subdividing groups into different what you perceive the life stage to be is that if you can achieve that so, for example, all the people kind of whether it be 93 or 65 who are a bit more active but perhaps have just retired making that transition but, you know, they’ve just given up a volunteering role whether a group for them not saying it as well as I did last time but whether they might be better in a group and then other people who’ve got more kind of physical challenges and, erm, they’re at a different life stage or experiencing life then they might be better’. (Facilitator 1)*

One facilitator suggested that individuals who are unable to contribute effectively to the group could be screened out – this was said with a particular participant in mind who was disruptive and anti-social during group sessions:

*‘I mean it would certainly need to be looked at in terms of, you know, whether that particular individual is (A) going to benefit from attending or (B) if everybody else is going to benefit from them being a part of it because that’s the whole point, everybody around that table need to benefit from everybody else’. (Facilitator 4)*

While other facilitators made suggestions around the information that is provided to potential participants prior to recruitment, one facilitator suggested that potential participants should be provided with standardised information about what the group entails, so that some aspects are not over emphasised.

*‘I can imagine things that’ll get over emphasised (by a study team member when trying to recruit a participant) that look a little bit more like this, oh! but I can see this person really wants to meet people so I’ll sell that, but then I might not say that as much, and I don’t know that’s easy for me to speculate, erm, but I just wonder about that and I wonder whether there could be an extra kind of screening checklist to go through and to tick or to, you know, I’ve just got to make you aware that this is, this is what it’s about’. (Facilitator 1)*

## Discussion

### Principal findings

In the three studies discussed in this article GP mail-outs were successful methods of recruiting the numbers of participants required for large-scale, preventative, lifestyle intervention studies. In the Lifestyle Matters RCT, interviewed participants suggested that mail-outs should contain more information about what taking part in the study would involve. Other recruitment strategies including referral by district nurses and other health professionals, or using media and posters, made a trivial contribution to recruitment as previously reported by the FiT study [16]. Community engagement was successful in a non-randomised feasibility study, but not in the subsequent RCT of the same intervention.

GP mail-outs for both RCTs described in this paper resulted in a low yield of consenting participants (under 3%). Compared to other studies reporting recruitment via GP mail-outs, this figure is moderate; 7% of potential participants that were approached for involvement in the FiT study consented to participate, and 0.04% in the Booster study [15, 16]. These recruitment rates are part of an expected range of recruitment rates that empirical studies demonstrate are lower for prevention RCTs than for therapy RCTs [31].

Interviewed participants in the Lifestyle Matters RCT were happy having received a letter from their GP regarding the trial; but this would be expected as the majority of participants were recruited via this method. A few participants did speak of confusion on receiving such a letter; such a reaction has been documented in another trial [32]. Participants and facilitators felt that individuals who would most benefit from the intervention were not recruited to the study. The apparent socialisation and confidence of those who took part could have been due to the recruitment methods used; interested participants had to opt in to the study and contact the research team. Such an action requires a certain level of confidence, thus resulting in possible volunteer bias. The source and impact of such bias is currently under-researched in trials of behavioural interventions; the effect of participation itself may interact with such interventions [33]. It was hoped that district nurses and community engagement would allow the targeting of those who were isolated and would benefit most from the intervention. Instead, the use of GP mail-outs in the Lifestyle Matters RCT appeared to have resulted in a sample that was not as isolated as the intervention facilitators expected. Despite this, facilitators still witnessed improvements in participants, despite not viewing them as isolated individuals.

Several interviewed Lifestyle Matters RCT participants felt that direct contact with GPs would be the most beneficial method to recruit, although, as far as we are aware, this was not achieved in the studies we undertook. However, other studies have found difficulty in this type of recruitment due to GPs’ levels of understanding of research not being sufficient, time constraints, issues with introducing research to vulnerable populations, or due to emotional concerns that the GPs may have, such as worries over the patients’ eligibility [34, 35]. In other cases GPs can be overly enthusiastic to recruit, resulting in patients being recruited who are not eligible to participate [18].

Other primary care staff can be instrumental in identifying study participants, particularly groups such as older vulnerable people. District nurses may have acted as ‘gatekeepers’ to participants being recruited to the studies; gatekeepers may act paternalistically once they have decided that the experimental intervention is beneficial, potentially not offering the trial to clients who were the target population due to concerns around vulnerability and randomisation outcome (i.e. allocation to the control group) [36]. In addition, it seemed evident during the recruitment phases of the trials that recruitment of patients to a preventative clinical trial was not a priority for the health care services.

### Strengths and weaknesses

This paper presents data from three studies that recruited participants with similar characteristics. It adds empirical observations to the theories that prevention studies have comparably low recruitment rates and that recruitment by targeted mail-outs following database searches are more efficient than opportunistic referral by health professionals. Much of the data derive from a single UK region and the two large trials were conducted from the same trials unit, although we reference comparable studies conducted elsewhere. Only one of the three studies included qualitative data of relevance to this topic.

### Recommendations for researchers and commissioners

We recommend the use of GP mail-outs for large-scale RCTs as a time-efficient method of recruiting participants. Such mail-outs should include relevant information in order for the individual to understand the research project and nature of the intervention and/or control arm and should make it clear that the communication is from the individual’s GP. Researchers should be wary of the potential volunteer and participation biases that may cause certain populations to be under represented when using recruitment methods that require potential participants to actively make contact with the research team [37]. To reduce volunteer bias it may be necessary for potential trial participants to have direct contact with clinicians. To achieve this, it may be effective to ‘buy out’ recruitment capacity among health professionals who serve hard-to-reach and vulnerable populations or obtain honorary contracts for research nurses to work more effectively with such services. In addition to monetary mechanisms, it is pertinent to ensure clinicians ‘buy in’ to the research – they should understand the need for the study and the need for randomisation in conditions of clinical equipoise, given that social interventions can cause harms to target populations [38]. Training of health care staff may aid recruitment of vulnerable populations to clinical trials; training strategies are required that orientate staff towards perceiving recruitment as ethical and rational given therapeutic uncertainty [39]. In light of the increasing quantities of applied health research being undertaken, plus the significant clinical involvement required to test psychosocial interventions, it is difficult to commit resources to recruitment activities at times when health services are struggling to meet demands for routine clinical services.

The lack of relatedness in terms of participant recruitment between the feasibility study and the Lifestyle Matters RCT adds to a growing body of research around issues around answering important methodological questions in feasibility studies [40]. The feasibility study was not randomised; the addition of the control (usual care) arm to the Lifestyle Matters RCT may have caused difficulties recruiting isolated adults. The size of the studies was also a salient factor – the research team for the feasibility study were focussed on one location only and were able to identify a clear strategy for recruitment from that area which may not have been successful elsewhere; for example, in this case by linking with local health champions and providing taster sessions in well-used community venues. Lessons from the feasibility study helped design the main Lifestyle Matters trial, but research suggests that neither pilot nor feasibility studies can or should be used to estimate recruitment rates to the main trial [41]. We add to the previous recommendations that qualitative research should be used alongside feasibility studies to assess the feasibility of recruitment techniques [42].

### Future research

More research is needed into the characteristics of volunteers who enrol and those who do not to better understand the reasons for non-participation so that this problem can be addressed [43]. Participation bias threatens external validity, making it hard to say how generalisable the findings are. There is a lack of evidence regarding the effect on recruitment of adding a control arm when transitioning from a single-arm feasibility study to an RCT; this requires further quantification.

The cost-effectiveness of different recruitment methods would be important to take into consideration in order to assess the most efficient method of recruitment. Huynh et al. undertook a systematic review to identify research that has been undertaken comparing the cost of different recruitment strategies, identifying that only two studies compared costs of recruitment, with monetary incentives in these studies costing more per patient recruited than direct contact with the patient [44].

In order to assess general recruitment method effectiveness, and cost-effectiveness, embedded trials could be undertaken that compare two or more recruitment methods within a study. Such an approach has been recommended by the Systematic Techniques for Assisting Recruitment to Trials (START) programme [45]. One embedded trial compared the use of a £100 incentive to attract socially deprived and elderly patients to the use of no incentive, finding that although it led to an increased patient response, it did not attract the more socially deprived patients [46].

Further research is required to identify effective recruitment strategies for vulnerable older adults. Participants recommended ‘snowballing’ in order to identify other participants, which may be an effective method of identifying older adults who are isolated and difficult to recruit. Other studies have tested this method, finding that snowballing (also known as respondent-driven sampling; RDS) was a cost-effective method of identifying individuals [47]. It needs to be established if such a method could recruit the number and frequency of participants required for a large RCT that aims to recruit participants over a long time-scale. In addition, opt-out methods are becoming increasingly acceptable in cluster trials where outcome data are routinely collected [48]. However, with the new General Data Protection Regulation (which replaces the Data Protection Act) coming into effect from May 2018, opt-out methods of consent have effectively been outlawed due to the requirement for informed consent to be ‘unambiguous’ and involve ‘clear affirmative action’ [49].

## Conclusions

Evaluations of prevention interventions recruit differently at scale and with the possibility of randomisation to a control condition. Research participants seem to desire direct contact with health care professionals before participating in a trial, but a lack of prioritisation by community health and social care workers, together with gatekeeping, means that targeting those most in need of such interventions is difficult. Further research is needed to understand models by which community health and social care professionals can be persuaded of the value of RCTs and to recruit to them. Research should combine elicitation of views that inhibit recruitment together with education strategies to enable it. Formal assessment of the cost-effectiveness of the resulting interventions could be assessed in nested RCTs.

## Abbreviations

6CIT: Six-item Cognitive Impairment Test
FiT: Food and Immunity Trial
GP: General practitioner
NHS: National Health Service
NIHR: National Institute for Health Research
PCRN: Primary Care Research Network
PPI: Patient and Public Involvement
RA: Research assistant
RCT: Randomised controlled trial
RDS: Respondent-driven sampling
REC: Research Ethics Committee
SF-36: 36-item Short Form Health Survey
START: Systematic Techniques for Assisting Recruitment to Trials
UK: United Kingdom
